# Review: Associations among goods, impacts and ecosystem services provided by livestock farming

**DOI:** 10.1017/S1751731118002586

**Published:** 2018-10-18

**Authors:** B. Dumont, J. Ryschawy, M. Duru, M. Benoit, V. Chatellier, L. Delaby, C. Donnars, P. Dupraz, S. Lemauviel-Lavenant, B. Méda, D. Vollet, R. Sabatier

**Affiliations:** 1 Université Clermont Auvergne, INRA, VetAgro Sup, UMR Herbivores, 63122 Saint-Genès-Champanelle, France; 2 UMR AGIR, INRA, Université de Toulouse, INPT, 31324 Castanet-Tolosan, France; 3 UMR SMART-LERECO, Agrocampus Ouest, INRA, 44000 Nantes, France; 4 PEGASE, Agrocampus Ouest, INRA, 35590 Saint-Gilles, France; 5 DEPE, INRA, 75338 Paris, France; 6 UMR SMART-LERECO, Agrocampus Ouest, INRA, 35000 Rennes, France; 7 Université Caen Normandie, INRA, UMR EVA, 14032, Caen, France; 8 BOA, INRA, Université de Tours, 37380 Nouzilly, France; 9 Université Clermont Auvergne, AgroParisTech, INRA, Irstea, VetAgro Sup, UMR Territoires, 63000 Clermont-Ferrand, France; 10 UR Ecodéveloppement, INRA, 84914 Avignon, France

**Keywords:** ecosystem services, food system, land use, sustainability, trade-offs

## Abstract

Livestock is a major driver in most rural landscapes and economics, but it also polarises debate over its environmental impacts, animal welfare and human health. Conversely, the various services that livestock farming systems provide to society are often overlooked and have rarely been quantified. The aim of analysing bundles of services is to chart the coexistence and interactions between the various services and impacts provided by livestock farming, and to identify sets of ecosystem services (ES) that appear together repeatedly across sites and through time. We review three types of approaches that analyse associations among impacts and services from local to global scales: (i) detecting ES associations at system or landscape scale, (ii) identifying and mapping bundles of ES and impacts and (iii) exploring potential drivers using prospective scenarios. At a local scale, farming practices interact with landscape heterogeneity in a multi-scale process to shape grassland biodiversity and ES. Production and various ES provided by grasslands to farmers, such as soil fertility, biological regulations and erosion control, benefit to some extent from the functional diversity of grassland species, and length of pasture phase in the crop rotation. Mapping ES from the landscape up to the EU-wide scale reveals a frequent trade-off between livestock production on one side and regulating and cultural services on the other. Maps allow the identification of target areas with higher ecological value or greater sensitivity to risks. Using two key factors (livestock density and the proportion of permanent grassland within utilised agricultural area), we identified six types of European livestock production areas characterised by contrasted bundles of services and impacts. Livestock management also appeared to be a key driver of bundles of services in prospective scenarios. These scenarios simulate a breakaway from current production, legislation (e.g. the use of food waste to fatten pigs) and consumption trends (e.g. halving animal protein consumption across Europe). Overall, strategies that combine a reduction of inputs, of the use of crops from arable land to feed livestock, of food waste and of meat consumption deliver a more sustainable food future. Livestock as part of this sustainable future requires further enhancement, quantification and communication of the services provided by livestock farming to society, which calls for the following: (i) a better targeting of public support, (ii) more precise quantification of bundles of services and (iii) better information to consumers and assessment of their willingness to pay for these services.

## Implications

In this review, we attempt to explain the trade-offs and synergies derived from livestock production and analyse their relevance or irrelevance across spatial scales in search of options for a more sustainable livestock sector in Europe. We conclude by highlighting key aspects that need to be explored to further enhance, quantify and reveal the services provided by livestock farming to society.

## Introduction

Livestock is a major driver of rural landscapes and economics, but it also polarises debate over animal welfare, impact of animal product consumption on human health and on the environmental footprint of livestock production, including greenhouse gas (GHG) emissions and the relative inefficiency of energy and protein conversion into animal products (Steinfeld *et al*., 2006; Aleksandrowicz *et al*., 2016). Conversely, the various services that livestock farming systems provide to society are often overlooked and have rarely been quantified. These positive effects have been captured by the concept of multifunctionality, which stresses that beyond its productive function, livestock farming also has many more ‘secondary’ effects or positive externalities (Groot *et al*., 2010; Swanepoel *et al*., 2010). At the interface between ecology and economics, ecosystem services (ES) have been defined as ‘the benefits people obtain from ecosystems’ (Millenium Ecosystem Assessment, 2005). Ecosystem services result from the positive value that stakeholders (i.e. the socio-system) assign to certain functions or ecosystem structures. For example, grassland-based farming systems preserve cultural landscapes linked to emblematic cattle breed and gastronomy heritage across Europe (Beudou *et al*., 2017; Vollet *et al*., 2017) and contribute to air and water quality, climate regulation and biodiversity conservation (van Oudenhoven *et al*., 2012; Rodríguez-Ortega *et al*., 2014; Lemauviel-Lavenant and Sabatier, 2017). The ES framework introduces new ecological and social dimensions into the design and management of agricultural systems (Zhang *et al*., 2007; Lescourret *et al*., 2015). Nonetheless, acknowledging the services provided by livestock farming should not hide the need to weight these services against their negative impacts. These ‘dis-services’ have been defined as nuisances for humans that result from either ecosystem functioning (Lele *et al*., 2013) or negative externalities of livestock such as habitat loss and nutrient runoff (Zhang *et al*., 2007). Dis-services will be further referred as ‘impacts’ throughout the manuscript.

The aim of analysing bundles of services is to chart the coexistence and interactions between the various services and impacts provided by agriculture. It leads to consider and analyse ‘sets of ES that appear together repeatedly’ across sites and/or through time (Raudsepp-Hearne *et al*., 2010). This is particularly challenging due to the positive (synergies), negative (antagonisms, trade-offs) and generally non-linear relationships among services. Highlighting synergies and antagonisms among services makes it possible to identify sources at the origin of potential associations among ES (Mouchet *et al*., 2014) and their variability according to livestock production areas and, finally, levers for enhancing the services provided by livestock farming. Beyond theory, the concept of bundles of services can be mobilised to detect trade-offs and find win-win options to inform policy pathways (Rodríguez-Ortega *et al*., 2014; Lescourret *et al*., 2015). The challenge is to offer stakeholders operational tools and levers for action to optimise the services provided by livestock farming. The social and cultural dimensions are often neglected due to the lack of available indicators (Plieninger *et al*., 2013; Beudou *et al*., 2017) although accounting for on-farm and indirect jobs, for example, is likely to modify system ranking (Röös *et al*., 2016). In this review, we attempt to explain the trade-offs and synergies derived from livestock production and analyse their relevance or irrelevance across spatial scales in search of options for a more sustainable livestock sector in Europe.

Literature that focusses on bundles of services is rare because most papers address only one or two ES (Tancoigne *et al*., 2014). Here, we review three types of approaches to investigate the associations among impacts and services provided by livestock. We follow the three successive steps proposed by Mouchet *et al*. (2014) (i) detecting ES associations, (ii) identifying and mapping bundles of ES and (iii) exploring potential drivers using prospective scenarios. The first section details some of the associations that occur among ES in ruminant systems, crop-grassland rotations and landscape mosaics, when a given management factor affects several ES at the same time. In the second section, we review recent attempts to map ES from landscape to country or EU-scales. We then show how accounting for the complete set of goods, impacts and services provided by livestock farming enlarges this questioning. In the third section, we discuss how prospective modelling scenarios have been simulated to forecast the consequences of a breakaway from current production, legislation and consumption trends. These scenarios are projections of possible futures for livestock farming in the context of climate change. We conclude by highlighting key aspects that need to be explored to further enhance, quantify and reveal the services provided by livestock farming to society.

## Detecting associations among ecosystem services in ruminant systems and landscape mosaics

**Figure 1:**
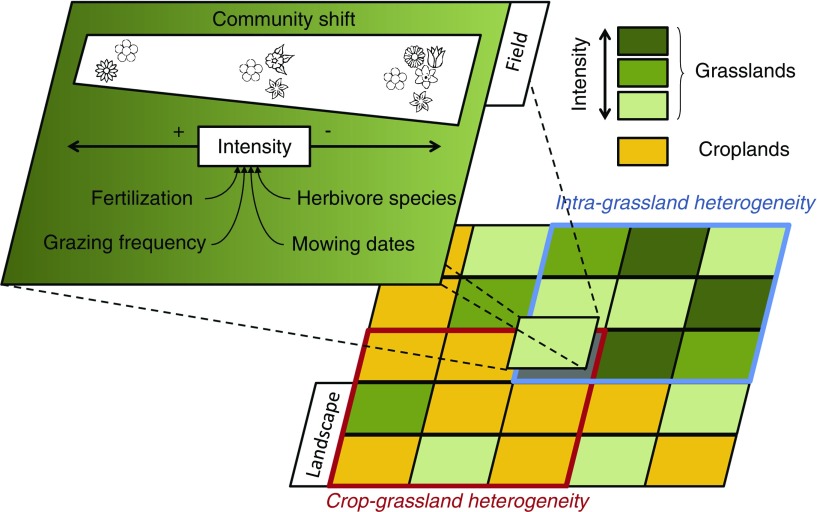
Multi-level effects of grassland management on biodiversity.

Many studies have analysed trade-offs and (less often) synergies between livestock production and other dimensions, especially a number of studies on the production *v.* ES dimensions in ruminant systems. Recent reviews (e.g. Fahrig *et al*., 2011), multi-site surveys (Lüscher *et al*., 2015) and meta-analyses (Scohier and Dumont, 2012; Herrero-Jáuregui and Oesterheld, 2018) have quantified the main effects of grassland management and landscape heterogeneity on biodiversity. They show how farming practices interact with landscape heterogeneity in a multi-scale process to shape grassland biodiversity (Figure 1). At the field scale, the main drivers of biodiversity are the type and intensity of management (e.g. grazing frequency, mowing dates, fertilisation) and the species of grazing herbivore. These drivers impact species richness in different taxa (vascular plants, arthropods: WallisDeVries *et al*., 2007). The effects of management on species richness are not limited to a reduction of the pool of species along an intensification gradient but also result in rapid shifts in community structure (Fleurance *et al*., 2016; Herrero-Jáuregui and Oesterheld, 2018). As a result, landscapes composed of grasslands managed in contrasting ways show a higher biodiversity than homogeneous landscapes (Fahrig *et al*., 2011). Conversion of some production lands into unmanaged or extensively managed land also leads to an increase in species richness for a range of taxa. Therefore, landscape heterogeneity is a key driver of biodiversity at higher levels of organisation (Figure 1) and, symmetrical, landscape homogenisation is a key driver of biodiversity losses. At the field scale, a decrease in year-round grazing intensity usually benefits biodiversity by an increase in sward heterogeneity (habitat heterogeneity hypothesis) and trophic resources (WallisDeVries *et al*., 2007; Herrero-Jáuregui and Oesterheld, 2018). However, it also strongly decreases production levels per unit area, which leads to a strong trade-off between production and biodiversity (Fleurance *et al*., 2016). Alternative management based on a fine tuning of the timing of grazing makes it possible to limit the negative effects on production; for example, temporary removal of cattle or sheep from some plots at flowering peak can have strong positive effects on grassland biodiversity while keeping grazing intensity at the farm level constant (Ravetto Enri *et al*., 2017). Such alternative management can be seen as a promising win-no lose option (for biodiversity and production, respectively) that demonstrates that one dimension of livestock sustainability can be improved without damaging another dimension. Within arable landscapes, grasslands (permanent but also temporary ones) favour biodiversity through two main mechanisms: (i) diversification of available habitats, which provides habitats suitable to grassland specialists (Figure 1); and (ii) provision of resources in specific periods of the year for generalist species that would suffer from the temporal discontinuity of resources provided by crop fields.

This multi-scale effect of grasslands on biodiversity can also be interpreted in the light of the ES that grassland biodiversity provides (Table 1). Biological regulation, pollination, water quality regulations and opportunities for recreation strongly benefit from grassland contribution to landscape mosaic. Input ES (i.e. the services provided by ecosystem to farmers; Zhang *et al*., 2007; Duru *et al*., 2018) such as soil fertility, biological regulations and erosion control, are highly dependent on farm-level heterogeneity, for example, on the length of pasture phase in crop-grassland rotations. This temporal dimension of land management may also lead to trade-offs between ES and production, as was revealed by Kragt and Robertson (2014). Their study was based on a farm system model that simulates production-possibility frontiers according to the length of the lucerne pasture phase in a grain crop-lucerne rotation. It revealed some initial ‘win-win’ possibilities between agricultural production and ES (increase in soil C sequestration and N-mineralisation, decrease in drainage). However, when more than 2 to 3 years of lucerne is included in the rotation, further environmental benefits come at a cost to agricultural production values. Production and various ES also benefit to some extent from the complementarity of grassland species with contrasting functional traits relative to resource acquisition and conservation (e.g. N capture, rooting depth) that enhances resource utilisation (Table 1). A 30% average increase of mixture annual yield compared to monocultures has been observed across a range of intensively managed European grasslands (Finn *et al*., 2013). Grass–legume mixtures are particularly relevant for forage production. Atmospheric N_2_ fixation by legumes increases N availability and thus decreases the inputs needed for production. Daily animal intakes of forage and digestion were also shown to be higher when fed on grass–legume mixtures (Niderkorn *et al*., 2015). The range of ES provided by grazed pastures, for example, C sequestration, also varies according to pasture management. Ecosystem services can indeed be impaired by intensification beyond a given stocking density threshold, which varies according to soil, climate and vegetation. Within extensively used grasslands, the main cycling elements, C, N and P, are naturally coupled by plant photosynthesis and soil microbial activity. Ruminants tend to uncouple the C and N cycles by releasing digestible C as CO_2_ and CH_4_, and by returning digestible N at high concentrations in urine patches. When intensifying pasture use, uncoupling activity of animals progressively offsets the C–N coupling capacity of the soil-vegetation system (Soussana and Lemaire, 2014); this leads to pasture degradation and to a decline in soil C sequestration, and an increase in nitrate leaching and nitrous oxide emissions to the atmosphere. After an initial win-win between livestock production and ES, livestock production thus increases at a cost to ES and then levels-off, whereas ES collapse.

**Table 1:** Overview of main ecosystem services (ES) provided by grasslands according to their functional diversity, length of pasture phase in the crop rotation and contribution to landscape mosaic: NE, no effect; from light + to high +++ effect (adapted from Duru *et al*., 2018)

	Functional diversity of grasslands	Grasslands in crop rotations	Grasslands within landscape mosaic
Production			
Forage production, flexibility in management	+++	+	NE
Input ES			
Biological regulations	+	++	+++
Soil fertility (nutrients, structure regulations)	++	+++	NE
Soil stability (erosion control)	NE	+++	+++
Pollination	+++	NE	+++
Other ES			
Water quality regulation	+	++	+++
C sequestration	+	+→ ++	NE
Moderation of extreme events: flood, running fires	NE	+	++
Opportunities for recreation	++	+	+++

Carbon sequestration provided by grasslands in crop rotations strongly varies according to the length of the pasture phase (+ → ++). Input ES are defined as the services provided by ecosystems to farmers, whereas other ES are the services provided by ecosystems to society.

Literature shows fewer studies on the relationships between agricultural production value and socio-cultural factors. An example of the trade-offs between production cost and the social dimension comes from animal welfare legislations that have determined the minimal area required per animal in livestock housing facilities. Synergies occur among animal welfare, individual performance (Coignard *et al*., 2013) and animal health (Bertoni *et al*., 2016). However, by increasing the area required per animal, these legislations have also increased production cost for a given level of product. Other synergies or trade-offs between production and socio-cultural dimensions remain largely overlooked. At farming system scale, first attempts have investigated relationships between production and employment (on-farm jobs and induced effects of the livestock sector on employment), contribution to social cohesion, agrotourism, educational value (farms that widen knowledge about plant and livestock species), cultural know-how and gastronomy with specific livestock product relating to local heritage (Plieninger *et al*., 2013; Beudou *et al*., 2017). Relationships between production and recreation (walking, hunting, etc.) or landscape aesthetic value are usually considered at wider scales (van Oudenhoven *et al*., 2012; see next section).

## Identifying and mapping bundles of ecosystem services and impacts

Several recent studies conducted from landscape (e.g. van Oudenhoven *et al*., 2012) to EU-wide scales (e.g. Maes *et al*., 2012) have evaluated and mapped ES across agricultural areas. These maps allow the identification of landscape areas where agroecosystem management has produced bundles of services that are more or less balanced among the different dimensions and thus target areas for policies that aim at a more sustainable agriculture future. The maps have revealed positive correlations between livestock density and crop production (and thus areas that are strongly oriented towards production), for example, in Denmark (Turner *et al*., 2014), although livestock density and percentage of land under crop production are unrelated to each other at the EU-wide scale (Maes *et al*., 2012). Trade-offs frequently occur between livestock density and most regulating (soil C sequestration, water conservation, erosion control) and cultural services (aesthetic, recreation and tourism) in all biogeographical areas (Maes *et al*., 2012; Turner *et al*., 2014). Grassland-based areas provide higher levels of regulating and cultural services when livestock density decreases (Maes *et al*., 2012; Van der Biest *et al*., 2014) and when grasslands are managed for higher functional diversity and landscape heterogeneity (Table 1; Rodríguez-Ortega *et al*., 2014). Although it is difficult to simultaneously reach high levels of production and ES, some areas provide more balanced bundles of services, for example, agricultural and recreational landscapes on the fringe of densely populated towns (van Oudenhoven *et al*., 2012; Turner *et al*., 2014).

However, these studies have several limitations. For example, Jopke *et al*. (2015) did not include animals fed indoors in their livestock density indicator calculated for all EU member states, and livestock density was thus no longer involved in any trade-off with regulating and cultural services. These results do not fit findings from Maes *et al*. (2012) and Turner *et al*. (2014) and reveal the extent to which selection of indicators can affect the outputs of a given study. Beyond this, the geographical externalisation of impacts (e.g. deforestation in Latin America for soybean production) is ignored in the mapping of ES across European agricultural areas. The numbers of livestock produced per grid cell are converted into Livestock Units (LU) whatever the relative proportion of local and imported feed resources in their diet. A second major limitation of some of these studies is that they aggregated production into a single indicator accounting for ‘food produced from agriculture’ (Palomo *et al*., 2014) or ‘total biomass production on agricultural land’, including short-rotation coppices for energy production in Kirchner *et al*. (2015). Even when a distinction is made, it remains incomplete, as in Turner *et al*. (2014) who, for Denmark, distinguished between livestock and crops but not between dairy cows and pigs. A third limitation is that correlations between services do not reveal any cause-and-effect relationship unless the drivers and the interactions between services have been made explicit. In most cases, the analysis relates to the simple spatial co-occurrence of services, whereas only spatial autocorrelations, clustering, repeatability or canonical analyses could define ES bundles and demonstrate causal relationships in the spatial clustering of services (Mouchet *et al*., 2014). Finally, the selection of indicators and how the study area has been divided into grid cells is constrained by available data sets. Maps thus chart the services provided by livestock farming systems at a coarse-grained scale that accounts for the effects of dominant socio-technical systems and fails to consider services provided by niche systems or that result from interactions between systems.

**Figure 2:**
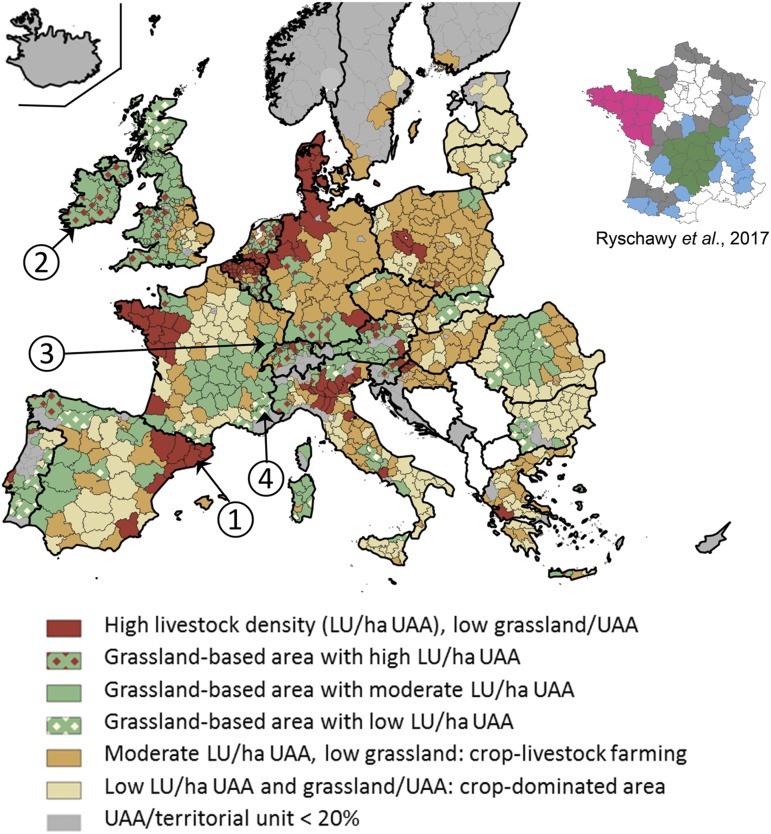
Typology of European livestock production areas based on Eurostat data 2010 at the NUTS3 (Nomenclature of Territorial Units for Statistics) level, or NUTS2 level for Germany, Belgium and the Netherlands (reproduced from Hercule *et al*., 2017). NUTS areas with high livestock density and little permanent grassland (in red on the map) cover 35.5 million ha across Europe; high-density grassland-based areas: 21.5 million ha; intermediate-density grassland-based areas: 67.5 million ha; low-density grassland-based areas: 23 million ha; crop-livestock areas: 110 million ha; and crop-dominated areas: 91 million ha (Dumont *et al*., 2018). Figures surrounded by a circle are the four case studies presented in Figure 3. Map of bundles of goods and services from Ryschawy *et al*. (2017) is embedded as a zoom. LU=livestock units; UAA=utilised agricultural area.

Ryschawy *et al*. (2017) enlarged the ES approach to better account for the specific contribution of the livestock sector to rural vitality (e.g. relative employment in farms, in R&D and the agrofood industry, stability of employment) and culture (e.g. gastronomy, heritage landscapes, emblematic breeds) that have to date largely been neglected (Plieninger *et al*., 2013). Indicators and data sources for assessing the level of each service at the Nomenclature of Territorial Units for Statistics 3 level (NUTS3) in France are given in Ryschawy *et al*. (2017). The authors distinguished between dairy, ruminant meat, monogastric meat and egg production and identified four types of bundles of services: a first type (in pink in zoom from Figure 2) provides a high level of provisioning services and employment opportunities in high livestock density areas but is negatively related to environmental services. A second type (in green) delivers a more multifunctional bundle and is based on grassland-based ruminant farming. A third type (in blue) is associated with cultural and environmental services in grassland-based landscapes, including High Nature Value areas, but with lower levels of provisioning services than in the two previous types. In the fourth type (in grey), livestock farming provides all types of services at the lowest levels because crops largely exceed livestock production.

In line with this short literature review, Hercule *et al*. (2017) used two simple and widely available criteria to map livestock production areas across Europe: livestock density (in LU per hectare of utilised agricultural area (UAA)) and the proportion of permanent grassland (*sensu largo*, i.e. including rangelands) within UAA. These two criteria allow accounting for the variability of bundles of services as they are related to all main system characteristics: animal species, feeding strategy, manure management, etc. Based on Eurostat data for 2010, Hercule *et al*. (2017) distinguished six types of livestock production areas (Figure 2). In line with Pfimlin *et al*. (2005), grassland-based areas were defined as those where permanent grasslands cover more than 40% of the UAA. High-density areas were defined as those with more than 1.2 LU/ha UAA, low-density areas had less than 0.4 LU/ha UAA and intermediate-density areas were in between. Nomenclature of Territorial Units for Statistics areas where UAA was less than 20% of the total area were excluded from the analysis because they were considered relatively unaffected by agriculture and only accounted for 4% of the European herd on 5% of the UAAs (Hercule *et al*., 2017). Serbia, Bosnia-Herzegovina, Macedonia and Albania were not considered in Eurostat 2010 and were therefore excluded from the analysis.

**Figure 3:**
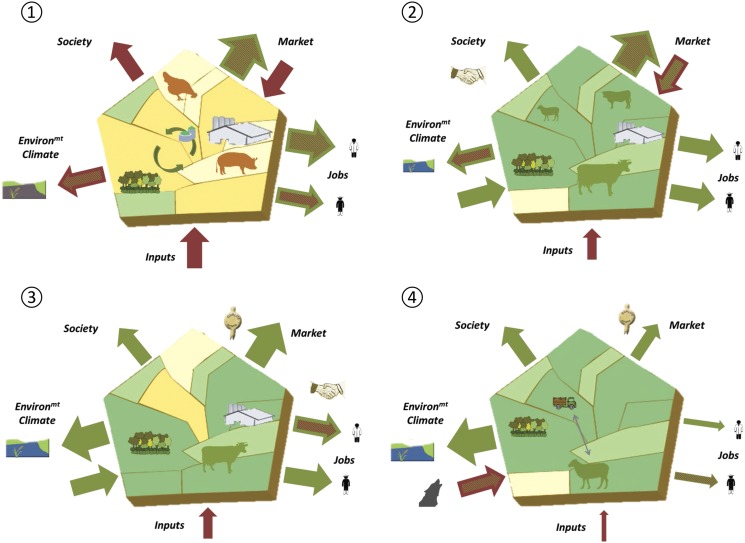
Bundle of services and impacts provided by livestock farming in four territories across Europe: (1) Catalonia (Dourmad *et al*., 2017), (2) Ireland (Delaby *et al*., 2017), (3) Franche Comté in northeastern French upland (Vollet *et al*., 2017), (4) Provence (Lemauviel-Lavenant and Sabatier, 2017). Duru *et al*. (2017) provided a full description of the ‘barn’ graphical approach. Within the pentagon, two shades of green account for permanent and temporary grasslands and two shades of yellow for the diversity of crop rotations. Grass-fed animals are in green, those fed with concentrate feeds in orange. Inward-pointing arrows represent market fluctuations, use of external input and ecosystem services (green) or dis-services (red). 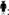
=on-farm jobs; 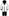
=indirect jobs; 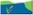 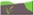
=good or poor water quality; 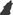
=predation risk; 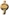
=quality labels for animal products; 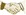
=collaboration between actors.

The map shown in Figure 2 is highly consistent with that proposed for France by Ryschawy *et al*. (2017), with contrasted bundles of services between types of livestock production areas. Areas with high livestock densities and little permanent grassland, as in Denmark (Turner *et al*., 2014), Brittany, Catalonia (Dourmad *et al*., 2017) and northern Belgium (Van der Biest *et al*., 2014), match the first type of Ryschawy *et al*. (2017). They account for 29% of the EU-wide livestock herd (mainly pigs, poultry and dairy cows) on only 10% of the EU-wide UAAs (Hercule *et al*., 2017). High average stocking densities, on average 2.17 LU/ha UAA, explain why manure management and reduction of local pollutions are major issues (Dourmad *et al*., 2017). Grassland-based areas (i.e. the second and third types of Ryschawy *et al*., 2017) have highly variable livestock densities. High-density areas, as in the Netherlands (van Oudenhoven *et al*., 2012) and Ireland (Delaby *et al*., 2017), account for 14% of the EU herd (mainly dairy cattle) on 7% of the UAAs, at an average stocking density of 1.66 LU/ha UAA. Intermediate-density areas with pasture-based livestock farming systems are multifunctional and provide many services to society (Rodríguez-Ortega *et al*., 2014; Kirchner *et al*., 2015; Vollet *et al*., 2017); they account for 18% of the EU herd on 18% of the UAAs. Low-density areas, as in Mediterranean grazinglands (Lemauviel-Lavenant and Sabatier, 2017), deliver many regulating and cultural services and account for 2% of the EU herd on 6% of the UAAs. Areas with intermediate livestock densities and few permanent grasslands have various dynamics, from integrated crop-livestock systems (Moraine *et al*., 2016) to areas where livestock declines due to competition with crops, olive groves, vineyards or fruit trees (Palomo *et al*., 2014). These areas account for 25% of the EU herd (balanced between ruminants and monogastrics) on 30% of the UAAs. Other agricultural areas are dominated by crops; they account for 8% of the EU herd on 24% of the UAAs. As detailed by Duru *et al*. (2017), the ‘barn’ framework can be used to simply represent the impacts and services provided by livestock farming. It illustrates how livestock farming interacts with its physical, economic and social environment along five interfaces (inputs, environment and climate, markets, labour and employment, and society) and provides bases for comparing livestock production areas. This is illustrated in Figure 3 for four case studies that cover a long gradient of intensification. Bundle of services is characterised by a high level of provisioning services in Catalonia that strongly contributes to EU pork-meat exports. Production is based on a very high use of external inputs to feed the animals, and has to deal with fluctuating prices for both feed and pork meat on international markets. A novel aspect of the barn framework is that it explicitly indicates impacts. Pollution risks contribute to the red outward-pointing arrow on the environment and climate interface for Catalonia, with also a negative pictogram for water quality (Figure 3). This contrasts with previous studies presenting low values for drinking water availability (Turner *et al*., 2014) or water quality (Ryschawy *et al*., 2017), from which it can be understood that there were detrimental effects of agriculture on water quality. In grassland-based areas, a decreasing gradient in livestock density from Ireland (Delaby *et al*., 2017), to Franche Comté in northeastern French uplands (Vollet *et al*., 2017) and Provence (Lemauviel-Lavenant and Sabatier, 2017) decreased the bulk quantity of livestock products, employment opportunities, use of external inputs and a number of environmental impacts (e.g. N-surplus per ha; Figure 3). Pasture-based systems benefit from input ES (Ireland, Franche Comté) but can be sensitive to drought and predation risk (Provence). Added value is created by quality labels in Franche Comté and Provence, with also collaborative agreements between farmers and cheese cooperatives in Franche Comté. The livestock sector benefits from a strong political support in Ireland where 80% to 90% of the milk and meat produced is exported on international markets. The barn framework thus graphically summarises the ecological and socio-economic aspects of livestock farming. Associated to the typology of European livestock production areas, it allows mapping impact and service bundles.

## Exploring potential drivers using prospective scenarios

Beyond the studies presented in the previous two sections that serve to detect, identify and map bundles of ES and impacts provided by livestock farming, and analyse their relevance or irrelevance across spatial scales, prospective scenarios (i.e. the projection of possible futures) evaluate global options to ensure food security in a more sustainable way. These scenarios simultaneously account for production and its environmental footprint, but also for population growth, trade of livestock products and human health. They simulate a breakaway from current production, legislation (e.g. the use of food waste to fatten pigs) and consumption trends (e.g. halving animal protein consumption across Europe) most often in interaction with climate change. Scenarios are not forecasts or predictions but offer a dynamic view of livestock futures by exploring trajectories of change that broaden the range of plausible alternatives (Mahmoud *et al*., 2009). Because these trajectories result from assumptions that are clearly stated, it becomes possible to discuss their likelihood and limits among stakeholders. Even, ES can be modelled and then be mapped or directly expressed as ES values per unit area (Mouchet *et al*., 2014). Scenarios are usually built through participatory approach for defining storylines. Then mathematical models are used to translate these qualitative narratives into a quantitative description of the corresponding nutrient and material flows, trade of livestock products, etc. However, it remains difficult to capture in mathematical models: (i) the diversity of livestock production areas; (ii) the social mechanisms behind food system transition, including consumer preferences and the volatility of opinion, although recent examples suggest crucial tipping points (e.g. slaughtering conditions) that could rapidly change consumer opinion; and (iii) the political and socioeconomic consequences (e.g. on rural vitality and landscapes with strong heritage value) of food system transition.

Most scenarios do simulate the effects of a decrease in animal protein consumption at either the global scale (Hedenus *et al*., 2014), EU-wide scale (Westhoek *et al*., 2014) or a national scale (Solagro, 2014; Röös *et al*., 2016). They successfully quantify how a decrease in livestock production would limit environmental impacts such as GHG emissions (Hedenus *et al*., 2014; Westhoek *et al*., 2014; Aleksandrowicz *et al*., 2016; Röös *et al*., 2016), N deposition (Westhoek *et al*., 2014) and land-use demand (Westhoek *et al*., 2014; Aleksandrowicz *et al*., 2016; Röös *et al*., 2016). Reductions in GHG emissions and in land-use demand are generally proportional to the magnitude of animal-based food restriction (Aleksandrowicz *et al*., 2016). However, some work has highlighted the existence of a lower limit for such environmental benefits. It was shown that minimal agricultural use of land in the Netherlands was achieved when 12% of Dutch protein intake was supplied by livestock products (Van Kernebeek *et al*., 2016) because the use of co-products from human food and forages from grasslands was optimised. The model showed that the land required for up to 25% of animal protein in the diet remained less than that needed to feed the same population eating a vegan diet.

Several scenarios have also been simulated to forecast the consequences of exclusively feeding livestock on permanent pastures, crop residues and food wastes that are not suitable for humans in order to limit feed-food competition. In Sweden, Röös *et al*. (2016) tested three livestock production scenarios that included a 20% reduction of total protein consumption. The two ‘extensive’ scenarios, in which adult cows were only fed forage decreased Swedish exports and required less land in Sweden and for feed imports. Model outputs revealed substantially lower environmental impacts on climate and N and P cycles for all scenarios compared with the current Swedish diet, although the effects remained higher than those recommended to remain within planet boundaries, that is, Earth’s biophysical thresholds which crossing would have disastrous consequences for humanity (Rockström *et al*., 2009). Doing this, these scenarios also decreased on-farm jobs, while the greater use of pesticides on crops would involve more contact with chemical substances for farm workers. Scenarios leading to a lack of dairy products or of eggs may result in low social and cultural acceptability of the diets. For France, the modalities of such transitions were discussed in the Afterres2050 scenario developed by the nonprofit Solagro association (Solagro, 2014). Hypotheses for change concerned diets, production methods and food waste. In line with the Agence Nationale de Sécurité Sanitaire de l’alimentation, de l’environnement et du travail (Anses, 2016) nutritional recommendations, Afterres2050 reduced total protein consumption by 25% (from 90 to 70 g/day) and the share of animal products from 40% to 25%, which led to increased pulse and cereal consumption. Food wastes were reduced by 60% and recycling was increased by the development of anaerobic digesters to produce energy. These hypotheses resulted in a halving of French animal production by 2050. Moreover, this scenario increased the relative contribution of organic, PDO (Protected Designation of Origin) and grassland-based systems and decreased the use of maize-soya beans to feed livestock. There would be substantially lower environmental and climatic impacts and a total of 125 000 jobs created in France by 2050 relative to a business-as-usual scenario. Such transitions in development models within the livestock sector would also modify the balance between on-farm *v.* indirect and induced jobs estimated with Input-Output Models. It is likely that new taxes, or at least suppression of incentives for equipment and inputs, would in turn favour on-farm jobs. Solagro (2014) estimated are a creation of 57 000 on-farm jobs and twice as many indirect jobs, which would compensate for the loss of 60 000 jobs in the agro-industrial sector.

At the EU-wide scale, halving animal protein consumption would achieve a 40% reduction in N emissions and a 25% to 40% reduction in GHG emissions while saving land for cereal exports, cultivation of pulses and energy crops or for nature purposes (Westhoek *et al*., 2014). Nitrogen-use efficiency would sharply increase from 18% to more than 40%. The intake of saturated fats would decrease by up to 40%, in line with nutritional recommendations. Another way to reduce N-surplus and the use of pesticides is the conversion of conventional to organic agriculture. A global food system model revealed that a 100% conversion to organic agriculture would need more land than conventional agriculture (Muller *et al*., 2017), which could be totally compensated by reductions of animal product consumption, of food waste and of feed-food competition. The same levers were analysed in another global scenario that assessed whether it would be possible to reach food security (assuming a global population of 9.7 billion people in 2050) while consuming animal products (van Zanten *et al*., 2016). Model outputs quantified three assumptions that are needed to produce a sustainable diet of ~21 g of animal-source protein per person per day, in line with nutritional recommendation: (i) decrease food waste from the current one third of food produced down to 10%, (ii) use the total area of permanent grasslands for milk and ruminant meat production and (iii) use co-products and food waste to feed pigs and poultry. Under these assumptions, the share of animal-source proteins would be one third from ruminants and two-thirds from pigs and poultry, although the third assumption would also require a change of EU animal feed legislation. If feeding pigs with cooked food waste was legalised in the EU and food waste was converted to animal feed at ~40% (as in Japan and South Korea), the land requirement of EU pork production could decrease by more than 20% compared with today’s land-use. Feeding pigs with food waste could replace 8.8 million tons of grains, which is equivalent to the annual cereal consumption of 70 million Europeans. Inclusion of cooked food wastes in pig diets is assumed not to affect meat quality (zu Ermgassen *et al*., 2016). However, such shifts in livestock feeding strategy would require safety precautions to check for food-waste contaminants and efforts to address consumer concerns over its acceptability.

Shifts in livestock feeding management together with a decrease in meat consumption can reduce GHG emissions from the livestock sector and thus its contribution to climate change (Popp *et al*., 2010; Aleksandrowicz *et al*., 2016). Inversely, there are studies that simulate the effects of climate change on the livestock sector, via its indirect effects on crop and grassland yields (Havlik *et al*., 2015). Overall, the proportion of livestock fed grass-based diets is expected to increase because grass yield would benefit more from climate change than crop yields (especially in North America and Southern Asia). In Europe, some regional changes in crop and grassland yields could lead livestock production areas to partly relocate. Climate change would favour mixed crop-livestock systems in Western Europe (Weindl *et al*. 2015) but the overall land-use pattern would remain stable (Havlik *et al*., 2015). In Austria, Kirchner *et al*. (2015) quantified several indicators for ES and economic development for different agricultural policy pathways that they combined with climate change scenarios. Changes in precipitation patterns were shown to increase the vulnerability of cropping systems. An increase in forage yield and stocking density on Alpine grassland would increase soil organic C sequestration and thus the contribution of Alpine grassland to climate regulation (Smith *et al*., 2005). Conversely, forage nutritive value would decrease as a result of direct (elevated CO_2_ decreases forage N content) and indirect (shifts in vegetation communities under elevated CO_2_) effects of climate change (Dumont *et al*., 2015).

## Research perspectives and policy pathways

In this article, we reviewed the information provided by three types of approaches and their main limits (Table 2) for assessing the positive and negative effects of livestock farming: (i) detecting ES associations at system or landscape scale, (ii) identifying and mapping bundles of ES and impacts, and (iii) exploring potential drivers using prospective scenarios. Whatever the scale or system considered, studies revealed a frequent trade-off between livestock production on one side and regulating and cultural services on the other. There are, however, various opportunities for win-win options that combine production, environmental and workload goals. In line with agro-ecological principles, these options are based on increasing forage autonomy in grassland-based systems, optimal use of co-products from human food to feed monogastrics (zu Ermgassen *et al*., 2016), increased functional landscape heterogeneity (Fahrig *et al*., 2011), and integration between farming systems (including crop or legume farms) that improves feed and manure management at both farm and landscape scales (Moraine *et al*., 2016). Win-win solutions are also more likely to emerge when they are the result of collective decisions that account for stakeholder preferences or ethical values, rather than situations where only individual interests and power relations prevail (Groot *et al*., 2010; Howe *et al*., 2014). Scenarios that simulate the effects of halving animal protein consumption confirm findings by Popp *et al*. (2010) that reducing meat consumption would strongly reduce GHG emissions from the livestock sector. Overall, strategies that combine a reduction of inputs, of the use of crops from arable land to feed livestock, of food waste and of meat consumption deliver a more sustainable food future (Hedenus *et al*., 2014; van Zanten *et al*., 2016; Muller *et al*., 2017). Livestock being part of this sustainable future requires further enhancement, quantification and communication of the services provided by livestock farming to society. This approach calls for: (i) a better targeting of public support, (ii) more precise quantification of bundles of services and (iii) better information to consumers and assessment of their willingness to pay for these services.

**Table 2:** Summary of the information provided by three methodological approaches reviewed in this article and of their main limits for assessing livestock impacts and services

	Accounts for	Main limits
Detecting ES associations at system or landscape scale	∙ Ecological processes (e.g. functional diversity of grasslands) ∙ Management and landscape characteristics ∙ Trade-offs and synergies among ES ∙ Legislation (area per animal in housing facilities)	∙ Site-specific quantification ∙ Sensitivity to system boundaries (imported feed not considered) ∙ Sensitivity to functional unit (performance/unit area or/unit product) ∙ Trade-offs between production and socio-cultural dimension largely overlooked
Identifying and mapping bundles of ES	∙ Diversity of livestock production areas ∙ Allows the identification of multifunctional areas ∙ Allows the identification of target areas with high ecological value or greater sensitivity to risks=> strategic landscape planning ∙ In some cases, climate change scenarios were considered (Kirchner *et al*., 2015)	∙ Niche systems hidden by the dominant socio-technical regime ∙ Livestock species not always distinct from each other’s and from crops ∙ Exported impacts not considered ∙ Using spatial co-occurrence of services does not reveal cause-and-effect relationships ∙ Evaluation constrained by spatial grain and indicator accuracy
Exploring potential drivers using prospective scenarios	∙ Decrease in animal protein consumption ∙ Exported impacts: land use, etc. ∙ Human health ∙ Climate change ∙ EU legislation on livestock feed ∙ In some cases, employment and cultural acceptability were considered (Röös *et al*., 2016)	∙ Scenarios and models usually do not account for the diversity of livestock production areas ∙ Mathematical models do not capture the social mechanisms behind food system transition (consumer preferences) ∙ Models do not capture the socio-economic consequences of scenarios (rural vitality, landscape heritage value)

These are in line with the three successive steps proposed by Mouchet *et al*. (2014) to investigate associations among ecosystem services (ES): (i) detecting ES associations, (ii) identifying and mapping bundles of ES and (iii) exploring potential drivers using prospective scenarios.

The Common Agricultural Policy (CAP) has long aimed to support livestock farming in areas where farming systems face pedoclimatic constraints that limit their competitiveness, which is also a way to support territorial vitality and to preserve biodiversity and landscapes with strong heritage value. Beyond this, the CAP also integrated some of the environmental dimensions of livestock farming. In high livestock density areas, policies such as the EU Nitrates Directive aim to regulate the local nuisances of water and air pollution from intensive farming. For example, the Nitrates Directive led to a decrease in N cycling disruption by clustering crop and livestock production into specific areas to optimise crop fertilisation (Turner *et al*., 2014; Dourmad *et al*., 2017). Conversely, agricultural policies hardly target global stakes such as climate change. The contribution of livestock to climate mitigation has not led to the fixation of farm emission thresholds that condition the attribution of public support. Agriculture was excluded from climate policies until the green payment for permanent grassland maintenance was introduced by the 2013 CAP reform. Such agri-environmental schemes are predominantly top-down and action-based, with farmers participating on a voluntary basis. The schemes only partly contribute to achieve sustainability goals (Kleijn *et al*., 2006) because they only weakly account for the regional diversity of bundles of services, stressed as important in this review. It thus appears essential to better target public support based on environmental susceptibility to pollution, habitat value, and an analysis of all the dimensions of bundles of services (including cultural services).

Payment for environmental services remains difficult due to a lack of relevant data or indicators. There have been proposals to quantify and pay for environmental services in organic farming and in the sheep-meat sector. Other studies have begun to quantify the negative externalities of European livestock such as habitat losses (Chaudhary and Kastner, 2016) and pollutions (Bourguet and Guillemaud, 2016) resulting from feeding livestock with crops and soya bean. All indirect costs and benefits must be systematically considered for a complete evaluation of bundles of services and impacts. Once these have been assessed, the question of political levers remains. Implementation of public support that aims to pay for ES requires a more precise quantification of the level of social benefits of a given service and the share of such benefits among local, regional, national and world community residents. Payment of penalties for high levels of dis-services runs up against the protectionist attitude of professional organisations and the fact that the European regulation does not encompass all the dimensions of livestock intensification. Common Agricultural Policy thus generally prefers the subsidy lever. Cross-compliance mechanisms that link direct payments to compliance by farmers with standards of Good Agricultural and Environmental Conditions provide basic protection for public goods and services. These are, however, not sufficient as revealed by the decline of insects and birds in and around agricultural areas (Hallmann *et al*., 2017).

Finally, it remains difficult to share the positive services provided by livestock farming (e.g. soil C sequestration, moderation of extreme events, etc.) with consumers and citizens so that they can develop their own system of consumption ethics. This calls to investigate their willingness to pay for the services provided by livestock farming. Apart from organic farming and PDO products, which are usually based on strict production specifications, most product labels are indeed vague, unverified and unverifiable (Treves and Jones, 2010). The labels may thus mistakenly claim that a product or production method offers an environmental, human health or animal welfare benefit, although legal instruments have recently made progress (http://eur-lex.europa.eu/eli/reg/2011/1169/oj). Beyond information to consumers, the bundle of services framework highlights the entire range of regulating, cultural and rural vitality services provided by livestock farming. By doing this, it allows the implementation of policy pathways that optimise livestock contributions to the global food system while remaining within planet boundaries.

## Software and data repository resources

Data used to propose a typology of European livestock production areas in Figure 2 are available online at https://doi.org/10.15454/O78MYF (Dumont *et al*., 2018). No software was generated at part of the outcomes of this literature review.
